# Differences between Vegetarians and Omnivores in Food Choice Motivation and Dietarian Identity

**DOI:** 10.3390/foods11040539

**Published:** 2022-02-14

**Authors:** Gahyun Kim, Jieun Oh, Misook Cho

**Affiliations:** 1 Department of Nutritional Science and Food Management, Ewha Womans University, Seoul 03760, Korea; kgh813@ewhain.net; 2 College of Science and Industry Convergence, Ewha Womans University, Seoul 03760, Korea; oje96@ewha.ac.kr

**Keywords:** vegan, vegetarian, omnivore, plant-based food, food choice motive, dietarian identity, consumer perception

## Abstract

Vegetarianism is on the rise worldwide and its importance is being emphasized in various ways, such as in its sustainability, environmental, food system, and ethical aspects. The purpose of the study is to identify motivations behind food choices and dietarian identity, to investigate the perceptions about plant-based foods, and to identify differences between vegetarians and omnivores. We conducted an online survey of 245 vegetarians and 246 omnivores. There was a significant difference between vegetarians and omnivores. In food choice motivations, vegetarians scored higher in the factors of ‘ethical concern’, ‘health’, and ‘convenience and price’, while omnivores responded higher in ‘sensory appeal’ and ‘weight control’ factors. In the dietarian identity, vegetarians scored higher in the ‘complex motivation’ and ‘strictness’ factors, while on the other hand omnivores scored higher in ‘out-group regard’ and ‘public regard’ factors. Although the reasons can be different, we confirmed that both vegetarians and omnivores are positive toward plant-based foods. Our results suggest that different strategies will be needed to promote plant-based food consumption to vegetarians and to omnivores.

## 1. Introduction

There is widespread consensus around the world that animal-product consumption needs to be reduced to avoid climate change, as livestock production accounts for a significant portion of greenhouse gas (GHG) emissions [1,2,3,4]. The production of livestock has a negative impact on the environment, disrupts biodiversity, and brings about unnatural climate change through the emission of greenhouse gases [5,6]. Previous studies have shown that although supply efficiency measures are important to reduce the negative impact of livestock production [7,8], it is also important to reduce overall meat consumption to meet global climate goals [5,9,10]. It has been reported that an increase in plant-based food consumption and a decrease in animal food consumption improve the sustainability of the food system [11,12,13].

A study [14] claimed that predicted changes in food consumption and production would substantially increase the impact of food systems on the environment and that, without specific measures, the risk of disrupting the main ecosystem processes could exceed the tolerable range. To resolve this, it has been proposed that the flexitarian diet, with its increased emphasis on plant-based foods, would be helpful. According to one study [15], as the positive effects of the vegetarian diet on climate change have been shown, campaigns such as “Meatless Monday” in the U.S. and the UK and “Veggie Thursday” in Germany and Belgium have been implemented. As such, the U.S. and Europe have made great efforts in promoting campaigns to raise public awareness of vegetarianism and to highlight its benefits.

As the benefits of vegetarianism have become more widely known, the number of vegetarians has steadily increased worldwide [16], but total meat consumption and per capita meat consumption have also concurrently increased [17]. In fact, because the intake of meat is still considered socially essential, it is important that social norms be established to acknowledge that the act of not consuming meat is something an individual can choose [18]. Accordingly, scholars have been conducting research to find intervention factors that increase plant-based food consumption and reduce animal food consumption [19,20,21,22,23].

Various studies have been conducted on the motivations behind vegetarianism. One study [24] conducted in 1998 suggested two reasons for choosing a vegetarian diet: health and ethics. Studies [25,26,27] in the early 2000s and a review study [28] in 2012 also showed that the two main vegetarian motivations are health and ethics. However, a review paper [29] conducted afterward and other studies [30,31] suggest that since the ethical motivations divided into concerns about the environment and animals, the representative vegetarian motivations are health, environmental, and animal concerns.

Several studies have investigated the differences between vegetarians and omnivores. A qualitative study [32] found that the four main motivations behind dietary choices for vegetarians were ‘animal welfare/rights’, ‘environmental issues’, ‘health/diet’, and ‘ethics and/or morals’, while those for omnivores, ‘taste and enjoyment’, ‘health/diet’, ‘ease of diet’, and ‘norms and socialization’, were investigated, showing that there is a difference, except for ‘health/diet’. Another qualitative study [33] explored barriers and facilitators toward meat substitutes in omnivores, vegetarians, and vegans. Other studies [34,35,36] compared vegetarian and omnivore diets in terms of nutritional quality. However, there are a few quantitative studies on the difference between vegetarians and omnivores in terms of food choices.

Dietarian identity is a comprehensive structure that reflects all aspects of self-awareness regarding a person’s choice of foods [37]. It is formed under the influence of sociocultural conditions, interpersonal relations, and personal preferences, whereas food choices are determined by the way people feel and act regarding what they eat [37]. The relationship between dietarian identity and food choice is thus bidirectional [38].

Food choice motivation refers to the reason or motivation of a consumer to choose a given food [39]. Various factors influence an individual’s food choices, such as the sensory properties of foods, food availability, social variables, health concerns, and increased awareness about the environment [40]. Understanding this motivation is crucial in innovations, campaigns, interventions, and policy developments regarding food consumption [39]. Overall, plant-based food substitutes will contribute to reducing meat consumption [41,42], and studies regarding consumer perception and behaviors toward plant-based foods, as well as personal dietarian identity and food choice motivation, will assist in creating and sustaining a niche for plant-based foods in the global market.

Various terms are used to refer to vegetarians, such as vegetarian, vegan, pescatarian, and pollotarian. Vegetarians are generally defined as individuals who do not consume meat, poultry, and fish [28,38]. Vegans, on the other hand, are defined as individuals who do not consume any animal-based foods at all [43]. Traditionally, vegans were regarded as a subgroup of vegetarians [28,38,44], but most vegans consider themselves different from other types of vegetarians [45,46]. To avoid confusion between these terms, the new term “veg*n” was coined by the VegForum to refer to both vegetarians and vegans [30], and the use of this term has recently increased among certain scholars [46,47,48,49]. To refer to both vegetarianism and veganism, the term “veg*nism” is used.

The objective of the study is to investigate and differentiate the dietary identities, food choice motivations, and personal expectations toward plant-based foods between vegetarians and omnivores. For this, the following hypotheses were made:

**Hypothesis 1** **.** *Food choice motivation may differ between vegetarians and omnivores.*

**Hypothesis 2** **.** *Dietarian identity may differ between vegetarians and omnivores.*

**Hypothesis 3** **.** *Dietary type may determine perception and behaviors toward plant-based food products.*

## 2. Materials and Methods

### 2.1. Participants

This study was conducted on a total of 491 individuals, comprising 245 vegetarians (vegan or vegetarian or semi-vegetarian) and 246 omnivores in the Republic of Korea. The criteria for the vegetarian participants were as follows: (i) individuals who perceive themselves as vegetarians (vegan, ovo-vegetarian, lacto-vegetarian, lacto-ovo-vegetarian, pescatarian, pollotarian, or flexitarian [50,51,52]); (ii) individuals aged 20–59 years who had agreed to participate; and (iii) individuals with experience of a vegetarian diet within the past two years.

To select the participants, non-probability sampling was used. To recruit individuals satisfying the criteria, a notice and the link to the questionnaire were posted on two online sites: Hanulvut Vegetarians (on the portal site NAVER), which has the largest number of members among the online communities, and the Korean Vegetarian Association. In addition, through acquaintances, the notice and the link to the questionnaire were shared with vegetarianism communities, such as the Vegetarianism Peace Alliance, and various social network sites (KAKAOTALK group chat rooms). The participants were those who read and understood the notice and participated in the questionnaire via the link (https://d8aspring.post-survey.com/open/?key=Og0UQzwF, last accessed on 20 May 2020); the completed questionnaires with the participant’s consent for participation were recorded.

The questionnaires for vegetarians were completed first; then, in the same proportions according to the age and sex of the vegetarian participants, the questionnaires for omnivores were completed. The online surveys of the omnivores and the vegetarians were carried out through DataSpring, Inc. (Seoul, South Korea) (https://www.d8aspring.com/ko, last accessed on 26 May 2020).

For ethical rigor, the study plan describing the purpose, contents, and methods was submitted to the Institutional Review Board of Ewha Womans University for review and approval (IRB No. Ewha-202002-0011-03). The survey was conducted from May 14, 2020 to May 26, 2020. The survey proceeded if the participants, who had read the study’s purpose and methods, subsequently agreed to participate.

### 2.2. Survey Tools

The questionnaires comprised 95 questions in total, including those for dietary type, perception toward plant-based foods, vegetarian experience, and demographic factors. To compare vegetarians and omnivores, the survey in this study contained two questionnaires. The questions regarding dietarian identity and food choice motivation were on a seven-point scale, whereas all the other questions were on a nominal scale. We used randomization when technically implementing FCQ and DIQ questionnaires on the web as a strategy to reduce the effectiveness of question-order bias, one of response bias. Therefore, the overall questionnaire order is the same for all respondents with FCQ, DIQ, perception of plant-based food products, dietary type, vegetarian experience, and demographic information; the order of questions in the FCQ and DIQ questionnaires was different for each respondent. The design of the entire questionnaire is presented in Table 1.

#### 2.2.1. Dietary Type

Although scholars have different descriptions of the specific types of vegetarianism, this study uses the categories presented by studies [50,51] to define the following eight types of diets: (1) full-time meat eater, (2) flexitarian, (3) pollotarian, (4) pescatarian, (5) lacto-ovo-vegetarian, (6) lacto-vegetarian, (7) ovo-vegetarian, and (8) vegan. Based on the criteria of studies [51,52], the eight diet categories were regrouped into four labels: (A) omnivore, (B) semi-vegetarian, (C) vegetarian, and (D) vegan. Label B has been called flexitarian in previous studies, but the term semi-vegetarian is used in this study to prevent confusion with the term in the eight-type categorization. In addition, although vegans were not specified in previous studies, they comprise label D in this study.

For dietary types, responses were made on nominal scales to the following two questions: eight categories (full-time meat eater, flexitarian, pollotarian, pescatarian, lacto-ovo vegetarian, lacto-vegetarian, ovo-vegetarian, and vegan) and four labels (omnivore, semi-vegetarian, vegetarian, and vegan) according to the categories and definitions provided in Table 2.

In addition, participants responded to DIQ’s dietary pattern question, the answers of which were presented in Section 2.2.3. This cross-check improved the accuracy of dietary type classification.

#### 2.2.2. Food Choice Questionnaire

The Food Choice Questionnaire (FCQ) with 44 questions of nine factors was used to assess food choice motivation. The FCQ comprised 36 questions of the original tool developed by a study [40] and eight questions of ethical factors added in a revision by a study [53]. The nine factors are health, mood, convenience, sensory appeal, natural content, price, weight control, familiarity, and ethical concern. The ‘ethical concern’ factor expanded by a study [53] consists of three detailed factors: ‘ecological welfare’, ‘political values’, and ‘religion’. The questions were in the form of, “It is important to me that the food I eat …”; the responses could be given on a seven-point scale from 1 (Not important at all) to 7 (Very important).

#### 2.2.3. The Dietarian Identity Questionnaire

The Dietarian Identity Questionnaire (DIQ) with 33 questions of eight factors, developed by the study [37], was used to assess dietarian identity. The eight factors are centrality, private regard, public regard, out-group regard, prosocial motivation, personal motivation, moral motivation, and strictness. The responses could be given on a seven-point scale from 1 (Strongly disagree) to 7 (Strongly agree).

According to the [37], before answering the 33 DIQ questions, participants were asked to choose one of the following six questions:

“I generally do not eat red meat.”

“I generally do not eat poultry.”

“I generally do not eat fish.”

“I generally do not eat dairy.”

“I generally do not eat egg.”

“I generally eat all of these food groups.”

The term ‘dietary pattern’, which appears repeatedly in the DIQ questions, refers to the dietary type of each person who answered this question.

#### 2.2.4. Perception toward Plant-Based Foods

The questionnaires were modified in accordance with the study purpose, with consideration of previous studies for investigating perception and behaviors regarding plant-based food products. The operational definition of “plant-based food products” as used in the questionnaires covered a broad range of plant-based foods but excluded completely natural or whole-plant-based foods. Hence, vegetables, fruits, whole grains, and beans without any processing or cooking were excluded. The definition of plant-based food products thus included traditional soybean-based processed foods (tofu, fermented soy, natto, tempeh, etc.), plant-based dairy substitutes, plant-based meats, plant-based bakery products, and plant-based snacks, and this study focused on these plant-based food products. The questions addressed general perception, purchase intention, and preferred labels.

#### 2.2.5. Vegetarian Experience

Only the vegetarian respondents were asked to respond to the following four questions related to the vegetarian experience: motivation, reason for continuation (single/multiple choice), duration of vegetarian diet.

Because the motivation might change when vegetarianism is maintained for a long time [30,37,44], the reason for continuation was also investigated. The participants were allowed to respond by choosing multiple reasons because vegetarianism is often maintained not for a single reason but for complex reasons [37,54].

#### 2.2.6. Demographic Information

Eight questions (sex, age, marital status, number of family members, family composition, occupation, education, and monthly household income) were used for the demographic information survey.

### 2.3. Data Analysis

SPSS Statistics 22.0 software was used to analyze the 491 questionnaires (245 from vegetarians and 246 from omnivores) collected through the online surveys. To identify the demographic characteristics of the participants and determine their perceptions of plant-based food products, frequency analysis was performed. For the 44 questions on the FCQ about food choice motivation and the 33 questions on the DIQ about the dietarian identity of vegetarians and omnivores, exploratory factor analysis (EFA) was performed to test the factor structure and question validity, and to evaluate the reliability. To determine the differences according to the vegetarianism or omnivorism dietary types, an independent t-test was performed.

## 3. Results

### 3.1. Demographic Characteristics of Participants

Table 3 presents the demographic characteristics of all the participants. The vegetarian dietary type, motivation, reason for continuation, and duration of vegetarian are presented in Table 4.

Among the 245 vegetarian participants, the self-identified vegetarianism dietary types were as follows: 124 vegans (50.6%), 9 ovo-vegetarians (3.7%), 15 lacto-vegetarians (6.1%), 24 lacto-ovo-vegetarians (9.8%), 37 pescatarians (15.1%), 13 pollotarians (5.3%), and 23 flexitarians (9.4%). When the types were identified based on the broader categories, 110 participants perceived themselves as vegans (44.9%), 53 as vegetarians (21.6%), and 82 as semi-vegetarians (33.5%). The self-identified types were different from those categorized on the basis of the participants’ responses and detailed criteria: 124 vegans (50.6%), 48 vegetarians (19.6%), and 73 semi-vegetarians (29.8%).

The motivations were as follows: health for 89 participants (36.3%), animal protection, 85 (34.7%), environmental protection, 37 (15.1%), religion, 15 (6.1%), influence of family or friend, 6 (2.4%), and other, 13 (5.3%).

The reason for continuation with the largest number of participants was animal protection at 95 (38.8%), followed by health, 82 (33.5%), environmental protection, 39 (15.9%), religion, 13 (5.3%), influence of family or friend, 5 (2.0%), and other, 11 (4.5%).

In the multiple choice for reasons for continuation, the results showed that animal protection was among the reasons for 177 participants (72.2%), environmental protection for 173 (70.6%), health, 164 (66.9%), influence of family or friend, 25 (10.2%), religion, 22 (9.0%), and other, 14 (5.7%). Three reasons were most frequently given by 94 participants (38.4%), followed by one reason by 72 (29.4%), two reasons by 48 (19.6%), four reasons by 30 (12.2%), and five reasons by 1 (0.4%).

The maintenance durations were as follows: ≥20 years for 22 participants (9.0%), ≥10 years and <20 years for 40 (16.3%), ≥5 years and <10 years for 17 (6.9%), ≥4 years and <5 years for 14 (5.7%), ≥3 years and <4 years for 20 (8.2%), ≥2 years and <3 years for 26 (10.6%), ≥1 year and <2 years for 47 (19.2%), ≥6 months and <1 year for 44 (18.0%), and <6 months for 15 (6.1%).

### 3.2. Food Choice Motivation

Table 5 presents the EFA results for food choice motivation. Among the total of 44 questions, those with factor loading ≤0.5, or commonality ≤0.5, or ≤2 items were excluded; based on a ≥1 eigenvalue, six factors were extracted from 34 questions. The explanatory distribution of the overall model was 66.529%, and the Kaiser–Meyer–Olkin (KMO) value to indicate the significance of the model was high at 0.910. The Bartlett’s sphericity test result was 10,506.299 with a significance probability of 0.000, which indicated significant correlations among the questions.

Factor 1 was labeled Ethical Concern because the responses were “Has been produced in a way that animals’ rights have been respected”, “Has been produced in a way that animals have not experienced pain”, “Has been prepared in an environmentally friendly way”, “Comes from a country in which human rights are not violated”, “Has been produced in a way which has not shaken the balance of nature”, “Is packaged in an environmentally friendly way”, and “Has been prepared in a way that does not conflict with my political values”. The eigenvalue of Factor 1 was 5.142 and the explanatory power was 15.123%.

Factor 2 was labeled Health because the responses were “Keeps me healthy”, “Contains no additives”, “Contains no artificial ingredients”, “Contains a lot of vitamins and minerals”, “Is good for my skin/teeth/hair/nails, etc.”, “Contains natural ingredients”, “Is nutritious”, and “Is high in fiber and roughage”. The eigenvalue of Factor 2 was 4.901 and the explanatory power was 14.416%.

Factor 3 was labeled Convenience and Price because the responses were “Is easy to prepare”, “Can be cooked very simply”, “Takes no time to prepare”, “Is easily available in shops and supermarkets”, “Is not expensive”, “Can be bought in shops close to where I live or work”, and “Is cheap”. The eigenvalue of Factor 3 was 3.840 and the explanatory power was 11.293%.

Factor 4 was labeled Mood because the responses were “Keeps me awake/alert”, “Helps me to cope with life”, “Cheers me up”, “Makes me feel good”, and “Helps me cope with stress”. The eigenvalue of Factor 4 was 3.267 and the explanatory power was 9.608%.

Factor 5 was labeled Sensory Appeal because the responses were “Smells nice”, “Has a pleasant texture”, “Looks nice”, and “Tastes good”. The eigenvalue of Factor 5 was 2.725 and the explanatory power was 8.014%.

Factor 6 was labeled Weight Control because the responses were “Is low in calories”, “Helps me control my weight”, and “Is low in fat”. The eigenvalue of Factor 6 was 2.405 and the explanatory power was 7.075%.

### 3.3. Dietarian Identity

Table 6 presents the EFA results for dietarian identity. Among the total of 33 questions, those with factor loading ≤0.5, or commonality ≤0.5, or ≤2 items were excluded; based on a ≥1 eigenvalue, four factors were extracted from 31 questions. The explanatory distribution of the overall model was 71.026%, and the KMO value to indicate the significance of the model was high at 0.966. The Bartlett’s sphericity test result was 14,018.825 with a significance probability of 0.000, which indicated significant correlations among the questions.

Factor 1 was labeled Complex Motivation because it was shown to comprise the five factors of centrality, personal motivation, prosocial motivation, private regard, and moral motivation in the original DIQ. The questions in detail were, “Following my dietary pattern is an important part of who I am”, “My dietary pattern has a big impact on how I think of myself”, “My dietary pattern defines a significant aspect of who I am”, “I follow my dietary pattern because eating this way improves my life”, and so on. The eigenvalue of Factor 1 was 9.675 and the explanatory power was 31.21%.

Factor 2 was labeled Out-group Motivation because it was shown to comprise the factors of out-group motivation in the original DIQ. The questions in detail were, “I judge people negatively for eating foods that go against my dietary pattern”, “Seeing people eat foods that go against my dietary pattern makes me upset or angry”, “I view people as less moral for eating foods that go against my dietary pattern”, “If I see someone eat foods that go against my dietary pattern, I like him or her less”, and so on. The eigenvalue of Factor 2 was 6.500 and the explanatory power was 20.967%.

Factor 3 was labeled Public Regard because it was shown to comprise the factors of public regard in the original DIQ. The questions in detail were, “People who follow my dietary pattern tend to receive criticism for their food choices”, “People who follow my dietary pattern are judged negatively for their food choices”, and “Following my dietary pattern is associated with negative stereotypes”. The eigenvalue of Factor 3 was 3.322 and the explanatory power was 10.715%.

Factor 4 was labeled Strictness because it was shown to comprise the factors of strictness in the original DIQ. The questions in detail were, “I can be flexible and sometimes eat foods that go against my dietary pattern”, “From time to time, I eat foods that go against my dietary pattern”, and “I would eat a food product that goes against my dietary pattern if I were to hear that it tastes exceptionally good”. The eigenvalue of Factor 4 was 2.521 and the explanatory power was 8.133%.

### 3.4. Comparison of Food Choice Motivation and Dietarian Identity between Vegetarians and Omnivores

To test H1, the first hypothesis of this study, t-tests were performed for the means of the FCQ factors based on dietary type, and the results are shown in Table 7. The testing of statistical significance with regard to food choice motivation between vegetarians and omnivores showed a significant difference for Ethical Concern, Health, Convenience and Price, and Sensory Appeal (significance level of 0.001), whereas for the Weight Control factor, a significant difference was found at the level of 0.01. The Mood factor, however, showed no significant difference. The results indicate that H1 can be partially accepted: food choice motivation differs between vegetarians and omnivores with the exception of the Mood factor.

Among the factors of food choice motivation, the score for Ethical Concern was significantly higher for vegetarians (5.98 ± 0.78) than for omnivores (4.39 ± 1.08) (*p* < 0.001), that for Health was significantly higher for vegetarians (5.64 ± 0.97) than for omnivores (5.13 ± 0.98) (*p* < 0.001), and that for Convenience and Price was significantly higher for vegetarians (5.91 ± 1.04) than for omnivores (5.39 ± 0.83) (*p* < 0.001). For Sensory Appeal, the score was significantly lower for vegetarians (4.73 ± 1.19) than for omnivores (5.39 ± 0.75) (*p* < 0.001). Likewise, for Weight Control, the score was significantly lower for vegetarians (4.46 ± 1.46) than for omnivores (4.81 ± 1.26) (*p* < 0.01). For Mood, no significant difference was found between vegetarians (5.56 ± 1.00) and omnivores (5.57 ± 0.82) (*p* = 0.951).

T-tests were also performed for the means of the DIQ factors between vegetarians and omnivores, and the results are given in Table 7. There was a significant difference for all of the tested factors constituting the dietarian identity; that is, Complex Motivation, Out-group Motivation, Public Regard, and Strictness (significance level of 0.001), and H2 were thus accepted.

Among the factors of dietarian identity, the score for Complex Motivation was significantly higher for vegetarians (5.59 ± 1.00) than for omnivores (3.27 ± 1.17) (*p* < 0.001), and the score for Strictness was also significantly higher for vegetarians (4.99 ± 1.59) than for omnivores (3.34 ± 1.04) (*p* < 0.001). For Out-group Motivation, the score was significantly lower for vegetarians (4.61 ± 1.50) than for omnivores (5.65 ± 1.30) (*p* < 0.001). For Public Regard, the score was also significantly lower for vegetarians (3.77 ± 1.40) than omnivores (5.54 ± 1.35) (*p* < 0.001).

### 3.5. Perceptions and Behaviors toward Plant-Based Food Products

The result of the crossover analysis of the perception toward plant-based food products based on dietary type showed χ2 = 64.739, with a significance probability of 0.000, indicating a significant difference in the perceptions toward plant-based food products between vegetarians and omnivores (Table 8). The number of vegetarians who were positive toward plant-based food products was 148 (60.4%), whereas 10 veg*ns (4.1%) were negative, 80 (32.7%) were ambivalent, and 7 (2.8%) answered no opinion. For omnivores, 160 (65.0%) were positive, 2 (0.8%) were negative, 30 (12.2%) were ambivalent, and 54 (22.0%) answered no opinion. To test H3, crossover analysis was performed for the purchase intentions with regard to plant-based foods based on dietary type, and the intention was shown to vary significantly according to dietary type (*p* < 0.001). For vegetarians, the most frequent response was Very high (90 participants, 36.7%), followed by High (57 participants, 23.3%). For omnivores, in contrast, the most frequent response was Slightly (85 participants, 34.6%), followed by Moderately (79 participants, 32.1%). The results of the crossover analysis for the preferred labels of plant-based foods based on dietary type showed χ2 = 221.098, indicating a significant difference at the level of 0.001. Most vegetarians were found to prefer the label, “Vegan foods” (149 participants, 60.8%), whereas most omnivores preferred the label, “Plant-based foods” (149 participants, 60.6%).

## 4. Discussion

To compare food choice motivation and dietarian identity between vegetarians and omnivores, and to investigate the differences in the perception of plant-based foods, an online survey was conducted on a total of 491 participants. Based on the results of the statistical analyses, all three hypotheses could be accepted.

Among the factors for food choice motivation, the scores for Ethical Concern, Health, and Convenience and Price were higher for vegetarians, whereas the scores of Sensory Appeal and Weight Control were higher for omnivores. The food choice motivation of vegetarians differed in specific ways from that of omnivores, and the results agreed with those of previous studies [26,55] to confirm that the difference did not result from cultural differences.

Among the factors for dietarian identity, the scores for Complex Motivation and Strictness were higher for vegetarians, whereas the scores of Out-group Motivation and Public Regard were higher for omnivores; these results coincided with those of one study [37]. For vegetarians, the high scores of Complex Motivation and Strictness may be indicative of a high value placed on dietary choice because these factors combine prosocial, ethical, central, and personal motivations. However, of the eight factors in the DIQ described by one study [37], only four factors were identified in this study, possibly because of participants’ personal and cultural differences. The Complex Motivation in this study includes five factors (centrality, personal motivation, prosocial motivation, private respect, and moral motivation) in the original DIQ. Future studies should recruit a greater number of participants from more diverse cultures.

Regarding the perceptions of plant-based food products, 148 vegetarians (60.4%) and 160 omnivores (65.0%) replied that they were “Positive”, indicating a higher proportion of individuals with a positive attitude. The result is in line with the results of a previous study [18], which reported people expect health promotion effects from reducing meat consumption and a study [56] that suggested health and environmental reasons are the driving forces behind the consumption of plant-based foods. However, 80 vegetarians (32.7%) in this study replied that they were “Ambivalent” toward plant-based food products and 54 omnivores (22.0%) responded with “No opinion”, results that differed between vegetarians and omnivores. The high proportion of vegetarians with an ambivalent opinion may be attributed to the concerns arising from the processing during commercialization. A lower level of processing means a comparatively higher level of eco-friendliness because less energy and packaging materials are required [57]. Moreover, in relation to health, vegetarians seemed to prefer foods with a “clean label” to indicate natural methods of production and minimal use of additives and processing [58].

With regard to the purchase intentions for new plant-based foods, a significant difference was found between vegetarians and omnivores. For vegetarians, the most frequent responses were Very High and High, whereas Slightly and Moderately were frequent for omnivores. Although the purchase intentions for new plant-based foods were higher in vegetarians, the intentions were not entirely negative in omnivores.

In considering the preferred labels for plant-based foods, most vegetarians were found to prefer the “Vegan foods” label and most omnivores were found to prefer the “Plant-based foods” label. We therefore consider that labeling with “Plant-based foods” would contribute to increasing the general consumption of these foods.

Despite the significant results, there are several limitations with this study. First, the traditional dietary habits in South Korea, mostly based on vegetables, are different from those in the West, but over time they became Westernized and meat consumption increased. Thus, a survey of the differences between generations is required but was not performed in this study, and future studies should investigate the differences between generations for comparative analysis. Second, because vegetarianism is one term that refers to people who eschew all animal-based foods, as well as those who selectively allow certain animal products, differences may be found in food choice motivations and dietarian identities among vegetarians. In this study, the type of vegetarianism was not specified in comparing vegetarians with omnivores, and further studies should separately analyze the specific vegetarian types for more accurate comparison.

## 5. Conclusions

This study is significant in having comparatively analyzed the food choice motivations and dietarian identities, as well as the perceptions of plant-based foods based on dietary type in Korean consumers, where the interest in vegetarianism and the number of vegetarians have increased.

All three hypotheses were accepted. In H1, concerning the food choice motivations, vegetarians scored higher in the factors of ‘ethical concern’, ‘health’, and ‘convenience and price’, while omnivores responded higher in the ‘sensory appeal’ and ‘weight control’ factors. In the case of H2, concerning dietarian identity, vegetarians scored higher in the ‘complex motivation’ and ‘strictness’ factors, on the other hand omnivores scored higher in ‘out-group regard’ and ‘public regard’ factors. Regarding H3, concerning the perceptions of plant-based foods, vegetarians and omnivores both had the highest percentage of ‘positive’ responses first, but there was a difference in that the second most common answers were ‘ambivalent’ for vegetarians and ‘no opinion’ for omnivores.

Based on the findings of this study, further studies should continue to investigate vegetarianism in South Korea with a multidisciplinary approach to contribute to the development of more sustainable dietary habits and cultures. In addition, through such studies, Korean society is anticipated to become healthier as more varied choices are allowed. What vegetarians value, in reality, is not just the aspects of dietary habits, but rather the overall lifestyle, such as minimizing their use of disposable products in daily life and promoting the use of products without animal testing. In the future, therefore, extensive research should be conducted to investigate the diverse aspects of vegetarianism in addition to dietary habits. Furthermore, cross-cultural studies of vegetarianism in various countries should be conducted.

## Figures and Tables

**Table 1:** Questionnaire design.

Item	*N* = 95	Variables	Scale
Food Choice Questionnaire [40,53]	44	Health, Mood, Convenience, Sensory appeal, Natural content, Price, Weight control, Familiarity, Ethical concern	7-point scale
Dietarian Identity Questionnaire [37]	1	Dietary pattern	Nominal scale
	33	Centrality, Private regard, Public regard, Out-Group regard, Prosocial motivation, Personal motivation, Moral motivation, Strictness	7-point scale
Perception toward plant-based food products	3	Perception, Purchase intention, Label	7-point scale, Nominal scale
Self-identifying dietary type [50,51]	2	Vegetarian type, Dietary type	Nominal scale
Vegetarian experience	4	Motivation, Reason for continuation (single/multiple choice), Duration	Nominal scale
Demographic information	8	Sex, Age, Marital status, Number of family members, Family composition, Occupation, Education, Monthly household income	Nominal scale

**Table 2:** Dietary types.

Category	Definition	Label	Designation
Full-time meat eater	Eats animal-based foods such as red meat, poultry, fish, dairy produce, and eggs.	Omnivore	Omnivores
Flexitarian	Consciously reduces meat intake, but eats meat now and then.	Semi-vegetarian	Vegetarians
Pollotarian	Eats no red meat, but eats fish, chicken, and other poultry.		
Pescatarian	Eats no red meat or poultry, but eats fish and shellfish.		
Lacto-ovo vegetarian	Eats no meat and fish, but eats eggs and dairy products.	Vegetarian	
Lacto-vegetarian	Eats no meat, fish, or eggs, but eats dairy products.		
Ovo-vegetarian	Eats no meat, fish, or dairy products, but eats eggs.		
Vegan	Eats no meat, fish, dairy products, or eggs, uses no products of animal origin.	Vegan	

Adapted from [50,51,52].

**Table 3:** Demographic profile of study participants.

Characteristics		Vegetarians (*n* = 245)		Omnivores (*n* = 246)	
		Frequency (*n*)	Percent (%)	Frequency (*n*)	Percent (%)
Sex	Male	68	27.8	70	28.5
	Female	177	72.2	176	71.5
Age (years)	20–29	73	29.8	72	29.3
	30–39	76	31.0	76	30.9
	40–49	60	24.5	60	24.4
	50–59	36	14.7	38	15.4
Marital status	Married	100	40.8	119	48.4
	Unmarried	136	55.5	125	50.8
	Other	9	3.7	2	0.8
Number of family members	1 person	71	29.0	31	12.6
	2 people	58	23.7	41	16.7
	3 people	57	23.3	57	23.2
	More than 4 people	59	24.0	117	47.6
Family composition	Alone	71	29.0	31	12.6
	With housemate	18	7.3	14	5.7
	Husband and wife	37	15.1	22	8.9
	Parents + children	110	44.9	164	66.7
	Other	9	3.7	15	6.1
Occupation	Students	32	13.1	22	8.9
	Office workers	84	34.3	122	49.6
	Full-time housewives	35	14.3	42	17.1
	Self-employed	23	9.4	16	6.5
	Freelancers	36	14.7	21	8.5
	Unemployed	16	6.5	20	8.1
	Other	19	7.8	3	1.2
Education	High school	18	7.3	38	15.4
	College	29	11.8	40	16.3
	University	161	65.7	142	57.7
	Graduate school	37	15.2	26	10.6
Monthly household income ($)	<1 M	16	6.5	8	3.3
	≥1 M and <2 M	24	9.8	19	7.7
	≥2 M and <3 M	58	23.7	32	13.0
	≥3 M and <4 M	41	16.7	59	24.0
	≥4 M and <5 M	40	16.3	52	21.1
	≥5 M	66	26.9	76	30.9
Total		245	100.0	246	100.0

**Table 4:** Vegetarian type, motivation, reason for continuation, and vegetarian duration of participants.

Descriptors		Vegetarians (*n* = 245)	
		Frequency (*n*)	Percent (%)
Vegetarian type (detailed/self-identified)	Vegan	124	50.6
	Ovo-vegetarian	9	3.7
	Lacto-vegetarian	15	6.1
	Lacto-ovo vegetarian	24	9.8
	Pescatarian	37	15.1
	Pollotarian	13	5.3
	Flexitarian	23	9.4
Vegetarian type (self-identified)	Vegan	110	44.9
	Vegetarian	53	21.6
	Semi-vegetarian	82	33.5
Vegetarian motivation	Health	89	36.3
	Animal concern	85	34.7
	Environmental concern	37	15.1
	Religious belief	15	6.1
	Influence of friend/family	6	2.4
	Other	13	5.3
Reasons to keep vegetarian (single choice)	Health	82	33.5
	Animal concern	95	38.8
	Environmental concern	39	15.9
	Religious belief	13	5.3
	Influence of friend/family	5	2.0
	Other	11	4.5
Reasons to keep vegetarian (multiple choice)	Health	164	66.9
	Animal concern	177	72.2
	Environmental concern	173	70.6
	Religious belief	22	9.0
	Influence of friend/family	25	10.2
	Other	14	5.7
The number of reasons to keep vegetarian	1	72	29.4
	2	48	19.6
	3	94	38.4
	4	30	12.2
	5	1	0.4
Duration of vegetarian diet	Less than 6 months	15	6.1
	6 months-1 year	44	18.0
	1–2 years	47	19.2
	2–3 years	26	10.6
	3–4 years	20	8.2
	4–5 years	14	5.7
	5–10 years	17	6.9
	10–20 years	40	16.3
	More than 10 years	22	9.0
Total		245	100.0

**Table 5:** Factor analysis of Food Choice Questionnaire (FCQ).

Factor	Item	Factor Loading	Commonality	Eigenvalue	Variance Explained (%)	Cronbach’s Alpha
Factor 1. Ethical Concern	FCQ 35. Has been produced in a way that animals’ rights have been respected.	0.879	0.822	5.142	15.123	0.913
	FCQ 34. Has been produced in a way that animals have not experienced pain.	0.879	0.817			
	FCQ 36. Has been prepared in an environmentally friendly way.	0.821	0.782			
	FCQ 40. Comes from a country in which human rights are not violated.	0.749	0.602			
	FCQ 37. Has been produced in a way which has not shaken the balance of nature.	0.747	0.726			
	FCQ 38. Is packaged in an environmentally friendly way.	0.742	0.601			
	FCQ 42. Has been prepared in a way that does not conflict with my political values.	0.690	0.505			
Factor 2. Health	FCQ 2. Keeps me healthy.	0.782	0.710	4.901	14.416	0.904
	FCQ 22. Contains no additives.	0.752	0.737			
	FCQ 24. Contains no artificial ingredients.	0.741	0.726			
	FCQ 1. Contains a lot of vitamins and minerals.	0.702	0.660			
	FCQ 5. Is good for my skin/teeth/hair/nails, etc.	0.674	0.559			
	FCQ 23. Contains natural ingredients.	0.668	0.694			
	FCQ 3. Is nutritious.	0.650	0.584			
	FCQ 6. Is high in fiber and roughage.	0.612	0.607			
Factor 3. Convenience & Price	FCQ 13. Is easy to prepare.	0.772	0.648	3.840	11.293	0.849
	FCQ 14. Can be cooked very simply.	0.762	0.628			
	FCQ 15. Takes no time to prepare.	0.698	0.585			
	FCQ 17. Is easily available in shops and supermarkets.	0.689	0.531			
	FCQ 25. Is not expensive.	0.689	0.584			
	FCQ 16. Can be bought in shops close to where I live or work.	0.652	0.532			
	FCQ 26. Is cheap.	0.567	0.545			
Factor 4. Mood	FCQ 10. Keeps me awake/alert.	0.743	0.704	3.267	9.608	0.849
	FCQ 8. Helps me to cope with life.	0.728	0.689			
	FCQ 11. Cheers me up.	0.701	0.683			
	FCQ 12. Makes me feel good.	0.661	0.620			
	FCQ 7. Helps me cope with stress.	0.567	0.578			
Factor 5. Sensory Appeal	FCQ 18. Smells nice.	0.748	0.638	2.725	8.014	0.791
	FCQ 20. Has a pleasant texture.	0.739	0.712			
	FCQ 19. Looks nice.	0.679	0.609			
	FCQ 21. Tastes good.	0.591	0.595			
Factor 6. Weight Control	FCQ 28. Is low in calories.	0.864	0.809	2.405	7.075	0.847
	FCQ 29. Helps me control my weight.	0.802	0.789			
	FCQ 30. Is low in fat.	0.693	0.669			

**Table 6:** Factor analysis of Dietarian Identity Questionnaire (DIQ).

Factor	Item	Factor Loading	Commonality	Eigenvalue	Variance Explained (%)	Cronbach’s Alpha
Factor 1. Complex Motivation	DIQ 5. Following my dietary pattern is an important part of who I am.	0.804	0.812	9.675	31.211	0.970
	DIQ 2. My dietary pattern has a big impact on how I think of myself.	0.804	0.734			
	DIQ 4. My dietary pattern defines a significant aspect of who I am.	0.777	0.775			
	DIQ 26. I follow my dietary pattern because eating this way improves my life.	0.772	0.642			
	DIQ 1. My dietary pattern is an important part of how I would describe myself.	0.755	0.694			
	DIQ 25. I follow my dietary pattern because I am concerned about the effects of my food choices on my own well-being.	0.754	0.582			
	DIQ 19. I view my dietary pattern as a way of making the world a better place for others.	0.750	0.794			
	DIQ 7. Following my dietary pattern is a respectable way of living.	0.744	0.579			
	DIQ 24. I follow my dietary pattern because eating this way is good for the world.	0.719	0.795			
	DIQ 21. I follow my dietary pattern because I want to benefit society.	0.710	0.783			
	DIQ 8. People who follow my dietary pattern should take pride in their food choices.	0.694	0.656			
	DIQ 3. A big part of my lifestyle revolves around my dietary pattern.	0.686	0.560			
	DIQ 22. I feel motivated to follow my dietary pattern because I am concerned about the effects of my food choices on other beings.	0.672	0.777			
	DIQ 30. I follow my dietary pattern because eating this way is the morally right thing to do.	0.662	0.769			
	DIQ 20. Concerns about social issues motivate me to follow my dietary pattern.	0.658	0.765			
	DIQ 28. I feel that I have a moral obligation to follow my dietary pattern.	0.552	0.768			
	DIQ 29. I am motivated to follow my dietary pattern because eating foods that go against my dietary pattern is immoral.	0.536	0.707			
	DIQ 23. I am motivated to follow my dietary pattern because I want to help others.	0.510	0.539			
Factor 2. Out-group Motivation	DIQ 13. I judge people negatively for eating foods that go against my dietary pattern. ^(R)^	0.865	0.780	6.500	20.967	0.936
	DIQ 14. Seeing people eat foods that go against my dietary pattern makes me upset or angry. ^(R)^	0.828	0.789			
	DIQ 12. I view people as less moral for eating foods that go against my dietary pattern. ^(R)^	0.819	0.746			
	DIQ 15. If I see someone eat foods that go against my dietary pattern, I like him or her less. ^(R)^	0.789	0.703			
	DIQ 16. It bothers me when people eat foods that go against my dietary pattern. ^(R)^	0.756	0.703			
	DIQ 17. Seeing someone eat foods that go against my dietary pattern makes him or her less attractive to me. ^(R)^	0.752	0.676			
	DIQ 18. People should feel guilty about eating foods that go against my dietary pattern. ^(R)^	0.724	0.684			
Factor 3. Public Regard	DIQ 10. People who follow my dietary pattern tend to receive criticism for their food choices. ^(R)^	−0.740	0.752	3.322	10.715	0.815
	DIQ 9. People who follow my dietary pattern are judged negatively for their food choices. ^(R)^	−0.700	0.679			
	DIQ 11. Following my dietary pattern is associated with negative stereotypes. ^(R)^	−0.640	0.590			
Factor 4. Strictness	DIQ 31. I can be flexible and sometimes eat foods that go against my dietary pattern. ^(R)^	0.850	0.766	2.521	8.133	0.770
	DIQ 32. From time to time, I eat foods that go against my dietary pattern. ^(R)^	0.810	0.699			
	DIQ 33. I would eat a food product that goes against my dietary pattern if I were to hear that it tastes exceptionally good. ^(R)^	0.696	0.722			

^(R)^ indicates a reverse-scored item.

**Table 7:** FCQ & DIQ differences between vegetarians and omnivores.

FCQ & DIQ Factors		Vegetarians (*n* = 245)	Omnivores (*n* = 246)	t-Value
		Mean (SD)	Mean (SD)	
FCQ factor	Ethical Concern	5.98 (0.78)	4.39 (1.08)	18.746 ***
	Health	5.64 (0.97)	5.13 (0.98)	5.784 ***
	Convenience & Price	5.91 (1.04)	5.39 (0.83)	−5.669 ***
	Mood	5.56 (1.00)	5.57 (0.82)	−0.061
	Sensory Appeal	4.73 (1.19)	5.39 (0.75)	−7.334 ***
	Weight Control	4.46 (1.46)	4.81 (1.26)	−2.820 **
DIQ factor	Complex Motivation	5.59 (1.00)	3.27 (1.17)	23.646 ***
	Out-group Motivation	4.61 (1.50)	5.65 (1.30)	−8.154 ***
	Public Regard	3.77 (1.40)	5.54 (1.35)	−14.229 ***
	Strictness	4.99 (1.59)	3.34 (1.04)	13.63 ***

** *p* < 0.01, *** *p* < 0.001.

**Table 8:** Overall perception, purchase intention, and preferred label for plant-based food products.

	Responses	Vegetarians (*n* = 245)		Omnivores (*n* = 246)		χ2 Value
		*N*	%	*N*	%	
Perception	Positive	148	60.4%	160	65.0%	64.739 ***
	Negative	10	4.1%	2	0.8%	
	Ambivalent	80	32.7%	30	12.2%	
	No opinion	7	2.8%	54	22.0%	
Purchase intention	Disagree strongly	11	4.5%	7	2.8%	100.703 ***
	Disagree moderately	7	2.9%	4	1.6%	
	Disagree a little	9	3.7%	14	5.7%	
	Normal	33	13.5%	79	32.1%	
	Agree a little	38	15.5%	85	34.6%	
	Agree moderately	57	23.3%	45	18.3%	
	Agree strongly	90	36.7%	12	4.9%	
Label	Plant-based foods	49	20.0%	149	60.6%	221.098 ***
	Vegetarian foods	43	17.6%	13	5.3%	
	Vegan foods	149	60.8%	19	7.7%	
	No label	4	1.6%	65	26.4%	

*** *p* < 0.001.

## Data Availability

The data presented in this study are available on request from the corresponding author. The data are not publicly available due to the institutional data policy.
